# Fully integrated ultra-sensitive electronic nose based on organic field-effect transistors

**DOI:** 10.1038/s41598-021-88569-x

**Published:** 2021-05-21

**Authors:** Daniil S. Anisimov, Victoria P. Chekusova, Askold A. Trul, Anton A. Abramov, Oleg V. Borshchev, Elena V. Agina, Sergey A. Ponomarenko

**Affiliations:** 1 Enikolopov Institute of Synthetic Polymeric Materials of the Russian Academy of Sciences, Profsoyuznaya str. 70, 117393 Moscow, Russian Federation; 2 Printeltech LLC, Profsoyuznaya str. 70, 117393 Moscow, Russian Federation

**Keywords:** Sensors, Sensors and biosensors, Molecular self-assembly, Electronic properties and materials

## Abstract

Modern solid-state gas sensors approaching ppb-level limit of detection open new perspectives for process control, environmental monitoring and exhaled breath analysis. Organic field-effect transistors (OFETs) are especially promising for gas sensing due to their outstanding sensitivities, low cost and small power consumption. However, they suffer of poor selectivity, requiring development of cross-selective arrays to distinguish analytes, and environmental instability, especially in humid air. Here we present the first fully integrated OFET-based electronic nose with the whole sensor array located on a single substrate. It features down to 30 ppb limit of detection provided by monolayer thick active layers and operates in air with up to 95% relative humidity. By means of principal component analysis, it is able to discriminate toxic air pollutants and monitor meat product freshness. The approach presented paves the way for developing affordable air sensing networks for the Internet of Things.

## Introduction

Detection of toxic gases, such as nitrogen oxides, hydrogen sulfide and ammonia, attracts many research activities due to increasing danger of air pollution related to industrial emissions, bumps and combustion engines^1^. These gases are also considered as disease markers in exhaled breath and can be used for non-invasive healthcare monitoring^2,3^. Moreover, decomposition of proteins is often accompanied by releasing amines and thiols, which can serve as markers of products freshness^4,5^. While comprehensive analysis of complex gas mixtures is usually made by benchmark gas chromatography, mass-spectrometry or optical methods, many practical tasks can be solved by catching mixture “fingerprint” mimicking mammalian olfaction^6^ with much more affordable array of semi-selective sensors of any type and pattern recognition algorithms^1–8^. The arrays based on metal-oxide-semiconductors^9,10^, gravimetric^11^ or conductive polymers^12^ sensors are known so far and have already been applied to manifold of sensing applications, while they are still costly, have limited sensitivity, insufficient selectivity or high power consumption due to heating^1,9,13^. Devices based on colorimetric sensor array consisting of a set of dye dots are promising for food industry, especially in smart packaging, but disposable components limit its implementation^5,14^. Future Internet of Things (IoT) smart sensor networks require sensitive, reusable, miniature, low-power and low-cost devices thus limiting widespread implementation of the technologies developed up to now^9^. Further sensitivity enhancement paves the way to automated determination of food freshness, which is the most ancient role of the sense of smell. This would alert many poison related diseases and drastically reduce unnecessary food waste, which is now up to 30% of all the food produced^15^.

OFETs are especially promising for gas sensing due to their architectural versatility, multiparametric detection, inexpensive production and low power consumption^16,17^. Decreasing the dimensions of their active layers to nanoscale have led to outstanding sensitivities: nano-wire^18,19^, nano-porous^20,21^ or monolayer^22–25^ based OFETs with a detection limit to some toxic gases in the ppb range have been reported. However, the biggest flaws of them are environmental instability and poor selectivity. Most of these sensors work only in vacuum, nitrogen or dry air and respond to a wide variety of interfering gases due to nonspecific interactions of the target analyte with the active layer. These reasons strongly limit benefits of the OFET sensors usage for real problems dealing with air mixtures containing water vapors and plenty of background gases that encourages a combination of cross-selective sensors into an array**.** Surprisingly, very few works devoted to OFET-based sensor arrays have been reported up to now (see Supplementary information, Table S1). Cross-selectivity in these arrays were achieved using different semiconducting materials in each OFET^26–32^. However, none of them operates in humid air or has sensitivity in the ppb concentration range. Moreover, single sensors in these arrays were in fact consequently measured one by one with a laboratory equipment.

**Figure 1:**
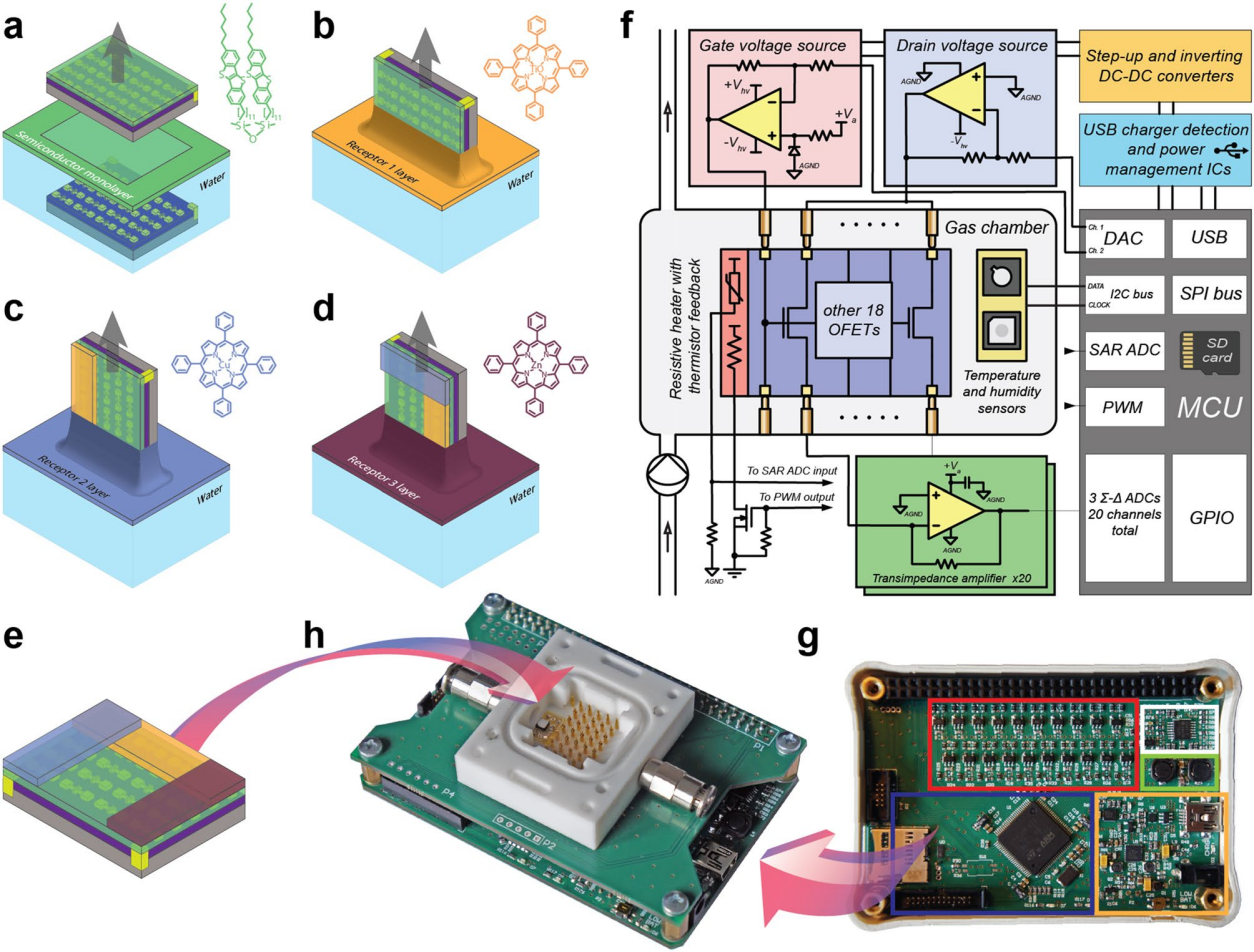
Schematic representation of the sensor array fabrication process and the electronic nose images. (**a**) Langmuir film deposition of organic semiconductor D2-Und-BTBT-Hex on a substrate with 20 transistors and its chemical structure. (**b**–**e**) Additional metalloporphyrin receptor molecules deposition and the corresponding chemical structures (TiO-TPP, Cu-TPP and Zn-TPP). (**f**) Device principal scheme. (**g**) Device component layout with analog part with an array of operational amplifiers shown in red, drain/gate voltage controlling operational amplifiers in white, DC–DC converters in green, digital part with a microcontroller and microSD-card slot in blue and USB charger/data transfer port and power management ICs in orange. (**h**) Photo of the device with sensor chamber with pogo pins array and reference T/RH sensor from the top.

In this work, we demonstrate the first fully integrated OFET-based electronic nose, which operates in air with up to 95% relative humidity and has down to 30 ppb limit of detection. It is capable to discriminate not only chemically different gases, such as nitrogen dioxide and ammonia, but also similar ones as hydrogen sulfide and ethanethiol. The ultrasensitive OFET-based sensor array with 2D nanoscale active layers was realized by Langmuir technique for organic semiconducting (OSC) monolayer deposition and its partial modification with different receptors on a single substrate to induce cross-selectivity (see Fig. 1a–e). The compact device (see Fig. 1f–h) simultaneously measuring response of the whole array was used to obtain all the data presented in this work. Its versatility was demonstrated by exceptional capabilities for environmental monitoring in wide humidity range and food freshness control.

### Sensor array fabrication

OFETs based on Langmuir-Schaeffer (LS) monolayer films prepared from amphiphilic benzothieno[3,2-b][1]-benzothiophene (BTBT) disiloxane derivative D2-Und-BTBT-Hex was used as elementary units of the sensing platform. This particular organic semiconductor was intentionally synthesized for Langmuir deposition methods requiring molecule amphiphility^33^. It contains hydrophilic siloxane anchor group, which improves monolayer film formation, and hydrophobic alkyl chains, which improve operational stability of the device at high humidity due to poor film wettability^26^_._ OFETs fabrication process is detailed in “Methods” and illustrated in Fig. 1a with the OSC chemical structure on the inset and Supplementary Fig. S1,2. The LS technique is scalable and thus suitable for low cost fabrication^34^. Moreover, it provides large area charge transporting 2D monomolecular layers of the OSC, which lead to outstanding sensing properties due to direct contact of the transistor conduction channel with the environment^35^. The OFETs demonstrate low hysteresis, high stability in the air and long operation time over one year due to oxidative and thermal stability of the BTBT-derivatives. Typical transfer curves of a single device and its partial degradation over one year at ambient conditions are shown in Supplementary Fig. S3. A stable response baseline that is crucial for electronic noses was achieved by suppressing competing bias stress-related charge trapping (see “Methods”). It was addressed by using a low surface trap density PMMA passivation layer on top of the SiO_2_ dielectric^36,37^ and by implementation of a pulsed gate measurement technique^38^.

Each substrate with 20 transistors was partially modified by subsequent Langmuir–Blodgett depositions of 3 additional receptor layers dividing it onto 4 groups of sensors (Fig. 1b–e): 3 groups of the OFETs with different receptor layers (orange, blue and violet areas) and a group of the non-modified OFETs (green area). Different porphyrinoids (see Figs. 1b–d and Supplementary Fig. S2), which are well known for tuning selectivity of gravimetric^11,16^, colorimetric^47^ and resistive^12,39^ sensor arrays, were used as receptors^40^. There is a large library of them, which enhance specific interactions with the analytes of choice depending on the metal atom in the coordination center^40^. For instance, titanyl-containing phtalocyanine (TiOPc) was found to be highly sensitive to nitrogen dioxide^41–43^, while copper-containing CuPc demonstrated lower response to NO_2_^44^. However, CuPc is sensitive to sulphur-containing molecules, such as SO_2_ and H_2_S^45^. Zn-containing receptors are suitable for detection of NO_2_ and NH_3_^46^, but have no response to H_2_S^47^. For better discriminative ability of the electronic nose, it is important to have varied cross-sensitivity over the sensor array, so each of the receptor chosen should have high sensitivity to several target analytes and have low sensitivity to remaining ones.

The other important factor for choosing the proper receptors was low film thickness and either uniform or spider-web like morphology providing many sorption sites. It was shown that gas absorption on organic films occurs mostly on domain boundaries and is strongly affected by the layer morphology^48,49^. For receptor layer deposition we have used Langmuir-Schaefer technique which provides thin layers required in order to keep monolayer OFETs high sensitivity and preserve its aligned crystalline structure due to the lack of organic solvent prior to the deposition stage. In this work we have used a set of Cu, Zn and TiO-tetraphenylporphyrins (TiO-TPP, Cu-TPP and Zn-TPP), having much better solubility than corresponding phthalocyanines, thus being suitable for solution processing. They have not only different metals in the coordinating centers but also various film morphologies presented in Supplementary Fig. S4, all affecting on the specific interactions with studied gases and improving the array discriminative ability^50^. However, the choice of particular receptors is not limited to these three metalloporphyrins and could be varied in accordance to the desired application.

### Non-modified OFETs sensor properties

Let us consider the sensor properties of a single OFET on the simplest case without a receptor layer. While general model describing the OFET sensing properties and response selectivity is still lacking, it is believed that a gas sensing mechanism is based on trapping of mobile charges in the conduction layer by adsorbed analyte molecules and related current change^51,52^. After the analyte exposure, the transistor transfer curve starts to shift because of related change of the charge carrier mobility, threshold voltage and contact resistance. OFETs are truly multiparametric sensors due to their nonlinear behavior, and in some cases consideration of independent OFET parameters as virtual sensors results in improved discriminative ability^53–55^, but a more general approach suitable to a wider range of the analytes is strongly required. It was previously shown that the highest sensitivity of the OFET-based sensors lies in a subthreshold voltage region^56^. However, in this work, we have focused on a saturation regime, which demonstrates the highest signal-to-noise ratio, and measured the current at fixed voltages instead of sweeping, which lead to less bias stressing and is easier for hardware implementation. The dependence of source-drain current in the saturation regime on variable gas concentration $[gas]$ and time can be expressed as the following:

1 $${I}_{SD}(t,\left[gas\right])= \frac{W}{2L}\mu {C}_{i}{({V}_{G}-{V}_{T})}^{2}$$

where $\mu$ and ${V}_{T}$ are the analyte dependent hole mobility and threshold voltage, respectively; ${C}_{i}$ is the gate dielectric capacitance per area; W/L are the channel width/length and ${V}_{G}$ is the applied gate voltage. Then the sensor response parameter measured at fixed voltages ${V}_{SD}$ and ${V}_{G}$ can be expressed through the current as the following:

2 $$Response (t,\left[gas\right])= \frac{I(t,\left[gas\right])-{I}_{0}}{{I}_{0}}*100\%$$

where $I\left[gas\right]$ is the steady state current value in the presence of the analyte at concentration [gas] and ${I}_{0}$ is the baseline value in its absence. The hole current and thus response parameter rises when the device is exposed to oxidative gases such as NO_2_ due to charge density increase and decrease when exposed to electron-donating gases such as NH_3_, EtSH and H_2_S, which is in accordance to previous OFET-based sensors studies^18–25^. It is important to note that this stands only for p-type devices with hole current, while opposite for n-type devices with electron conductivity^32,57^. For details of the gas sensing experiments, see “Methods”.

**Figure 2:**
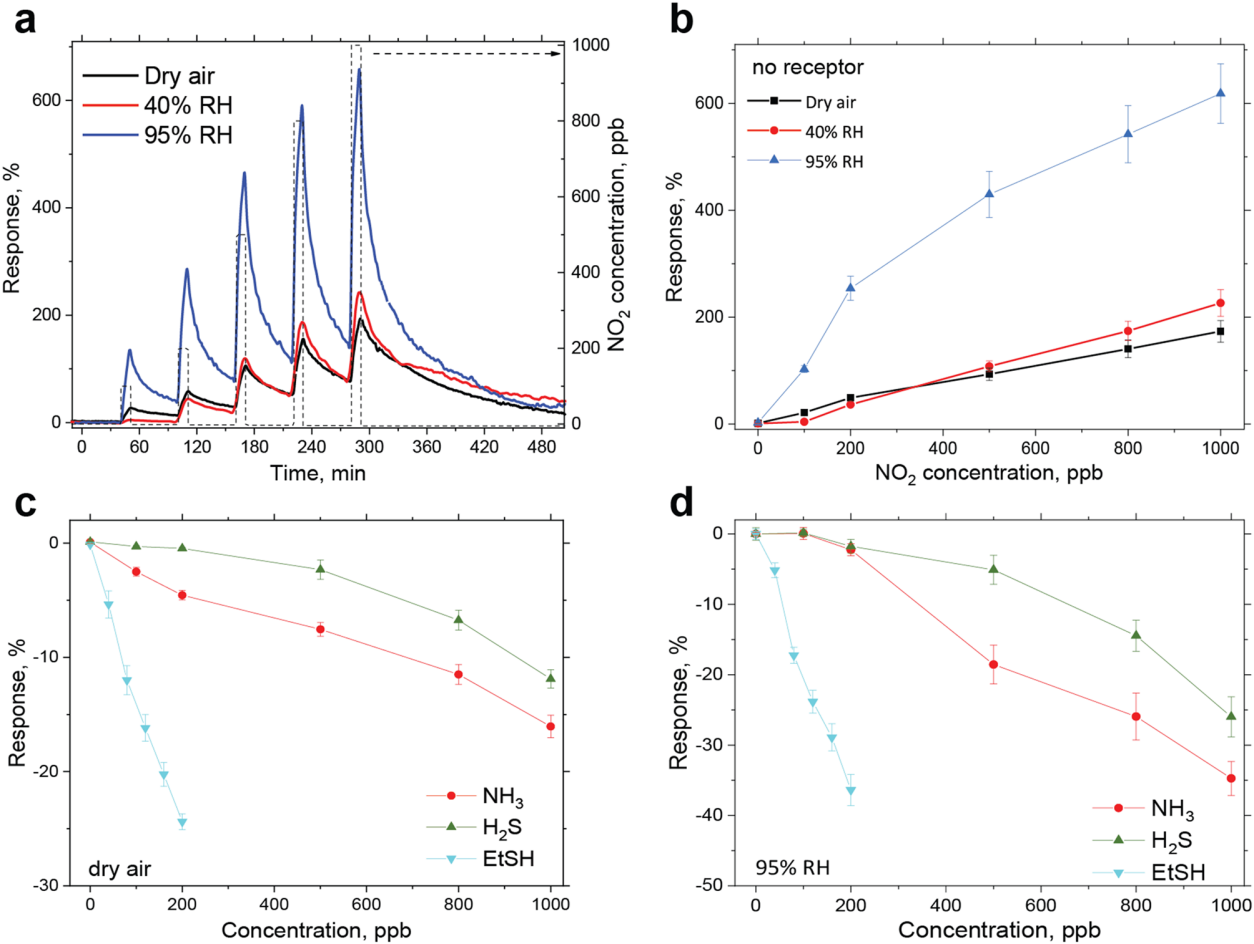
Properties of single OFET sensors without a receptor layer. (**a**) Dynamic sensor response to NO_2_ pulses at 100, 200, 500, 800, 1000 ppb concentration in dry (black) and humid air at 40% (red) and 95% (blue) relative humidity. (**b**) Corresponding response curves. (**c**,**d**) Response curves of the sensors for target analytes: NH_3_, H_2_S, Et-SH and NO_2_ in dry (**c**) and humid air at 95% RH (**d**). Mean values are presented with the standard errors.

Operation at high relative humidity (RH) is challenging for OFET-based sensors^26^ and is rarely studied for ultra-sensitive devices, while being a common circumstance for practical applications such as exhaled breath analysis or meat spoilage detection. However, the sensors presented in this work are stable and sensitive to the target analytes at high relative humidity in the background. The dynamic sensor responses to NO_2_ in dry and humid air at 40% and 95% RH reach up to 200–600% as shown on Fig. 2a with the corresponding response curves in Fig. 2b. The sensors response towards pure water vapors at different humidity level is just about 20 at 40% RH and 40% at 95% RH, respectively (see Supplementary Fig. S5a). Hence, response to 1 ppm of NO_2_ at 40% RH is just slightly higher as compared to the dry air, which could be described as superposition of the interactions of the sensing layer with water vapors and NO_2._ However, such superposition could not explain drastic sensitivity enhancement at 95% RH. It is suggested that at high humidity levels, water molecules on top of the sensor form a thin film^58^, inside of which the analyte molecules interact with water followed by dissociation to ions. Therefore, while “dipole-charge” interactions determine the sensor sensitivity to the target analyte in dry air, the stronger “charge-charge” interactions dominate at high relative humidity leading to the sensitivity enhancement. Nevertheless, the sensors have a limit of detection (LOD) to NO_2_ well below 50 ppb in the whole RH range measured (see Supplementary Table S2).

**Figure 3:**
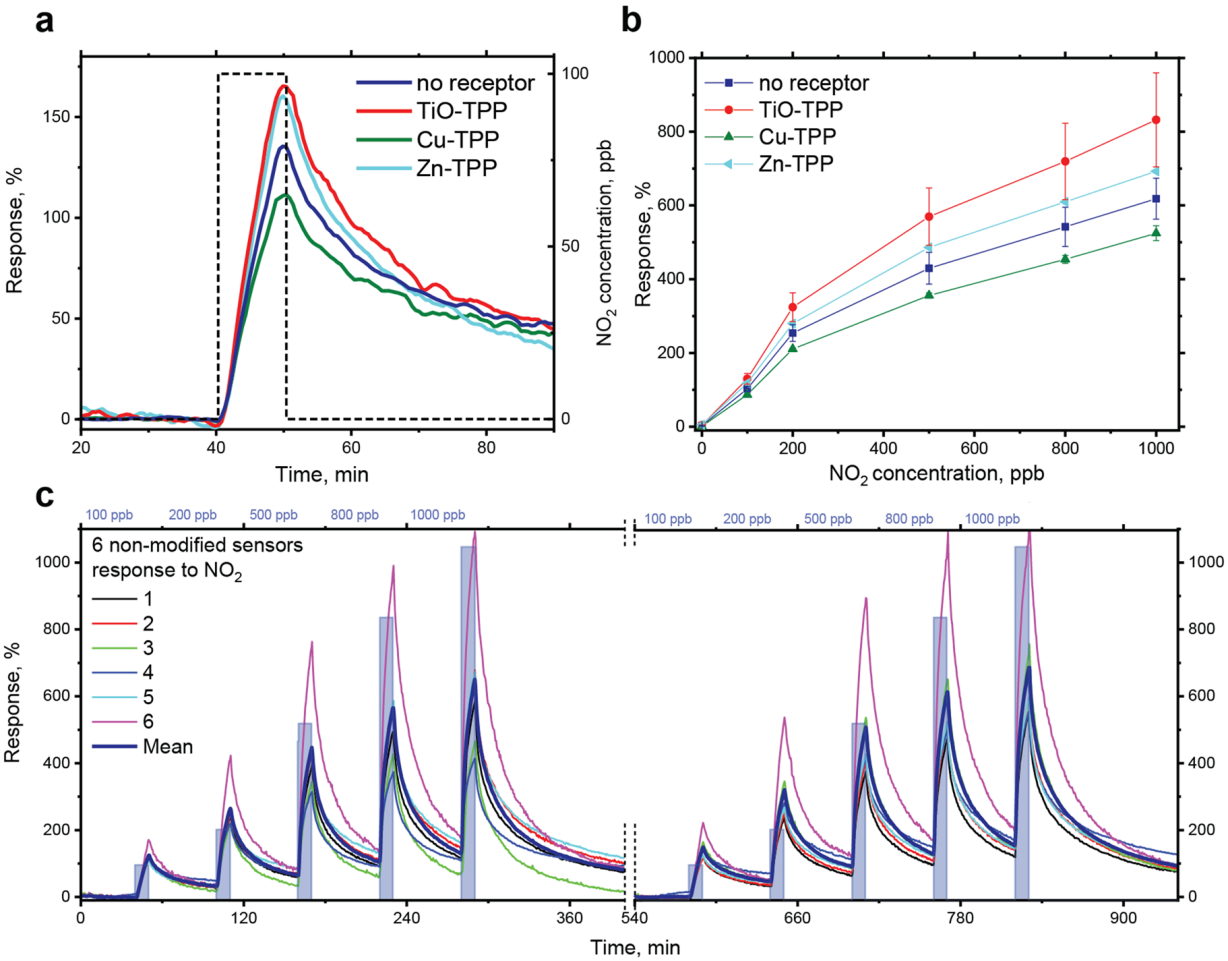
Effect of the receptor layer on the response selectivity, response reversibility and reproducibility. (**a**) Mean response of the sensor groups with different receptor layers: TiO-, Cu-, Zn-TPP and sensors without receptor layer towards 100 ppb of NO_2_ in humid air with 95% RH. (**b**) Response curves to NO_2_ at 95% for the non-modified OFET sensor and the devices with TiO-, Cu- and Zn-TPP receptor layers demonstrating their effect on the response selectivity. Mean values are presented with their standard errors. (**c**) Response-time (left axis) dependence for 6 non-modified OFET sensors of the array on a single substrate for 2 successive experiments in humid air at 95% RH (color lines) and its mean value (bold blue line) with NO_2_ pulses from 100 to 1000 ppb shown in light blue.

Cross-sensitivity to major interfering compounds was also checked and compared with the target analytes (see Fig. Supplementary Fig. S5b ). The sensitivity to alcohols and water vapor was found to be at least 3 orders of magnitude lower as compared to NO_2_ that can be caused by the presence of long hydrophobic alkyl chains in the OSC molecules used. This allows sensing the target analytes in the presence of many volatile organic compounds, CO_2_ and water vapors in the background, which is essential for a majority of real applications. Reproducibility of the response to NO_2_ was examined by repeated experiments with 6 identical sensors exposed to varied concentrations of NO_2_ twice in a row, which is presented in Fig. 3c. It can be seen that the response is fully reversible and a mean response of all non-modified sensors on one substrate to 100 ppb of NO_2_ at 95% RH gives 102% value with the standard error of 6%, n = 12. This is an additional advantage of having more sensors of each type, since single outliers lead only to a small deviation from the mean value. Enlarged response pulses over the time on a seconds timescale is shown in Supplementary Fig. S6 **.** The response towards ammonia, hydrogen sulfide and ethanethiol was also studied in dry and humid air with the response curves shown in Fig. 2c,d respectively**.** The sensors have responses of the opposite sign as compared to NO_2_ with the lower sensitivity to NH_3_ and H_2_S, while the sensitivity to Et-SH was comparable to those for NO_2_ with 40 ppb LOD. All sensitivities and LOD to the target analytes in dry and humid air are summarized in Supplementary Table S2 . They demonstrate linear behavior in the sub-ppm range, reversible response to all the gases investigated and LODs in the range of 30–470 ppb. However, despite the sensitivity difference, discrimination among these gases based on a single sensor response is almost impossible. The sensor properties of single OFETs with different receptor layers will be discussed in the next section.

### Sensor array properties

Each receptor layer on a substrate within the fabricated sensors array was represented by a subset of 3 to 6 sensors, according to the layout in Fig. 1e (for details see “Methods” section and Supplementary Fig. S2 ). All the measurements were averaged among the identical sensors on the substrate and two repeated experiments. Since many practical applications such as exhaled breath analysis and meat spoilage monitoring are dealing with gas mixtures at varied relative humidity, in this section we will focus on the sensor properties in the humid air, while all the data obtained for different humidity levels can be found in Supplementary Fig. S7,8.

The effect of the receptor layer on sensitivity to 100 ppb of nitrogen dioxide at 95% RH is compared in Fig. 3a, while full response-time dependence during the pulses of gases at varied concentration is shown in Supplementary Fig. S9 and corresponding response curves in Fig. 3b. It is clearly seen that Cu-TPP receptor layer suppress sensitivity to NO_2_ by 15%, while TiO-TPP and Zn-TPP layers enhance it by 35% and 12%, respectively, as compared to the response of non-modified OFET sensors to 1 ppm. Measurements details for all the gases investigated are presented in Supplementary Figs. S7-S8 and summarized in Supplementary Table S2. It should be noted that all the receptors interact with the analytes differently allowing capturing their peculiar features that is crucial for the array discriminative properties. Moreover, they keep the response linearity with LOD at 50–700 ppb range.

**Figure 4:**
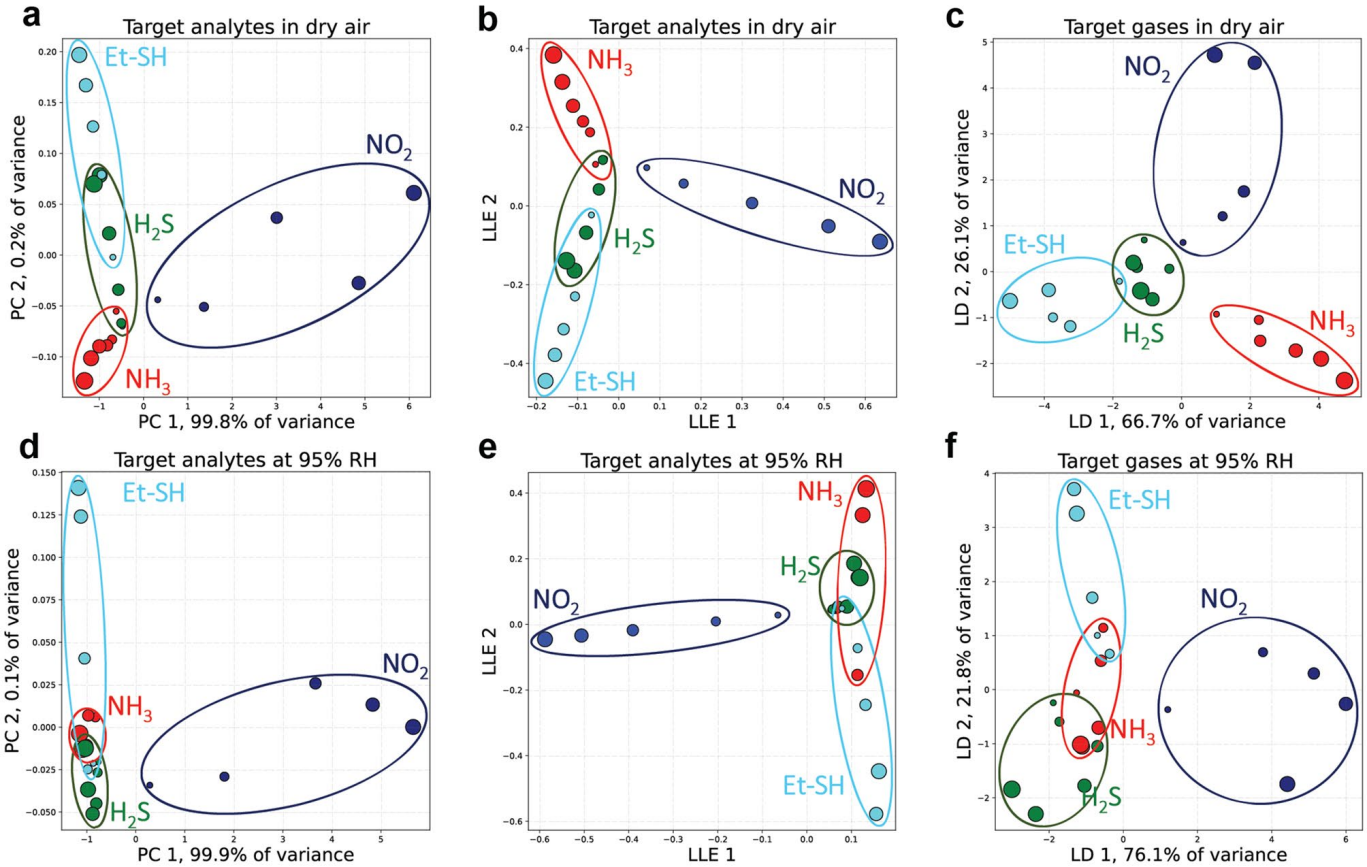
Sensor array discriminative abilities demonstration with dimensionality reduction algorithms in dry (top row) and humid (bottom row) air. (**a**,**d**) Principal Components Analysis (PCA). (**b**,**e**) Local Linear Embedding (LLE). (**c**,**f**) Linear Discriminant Analysis (LDA). Dot size corresponds to the concentration (not in scale; NH_3_ and H_2_S are presented at 100, 200, 500, 800, 1000, 1500 ppb, NO_2_ at 100, 200, 500, 800, 1000 ppb and Et-SH at 40, 80, 120, 160, 200 ppb concentrations). Dotted color ellipses include all the measurements related to a specific gas and border regions on the diagram.

To recognize the patterns corresponding to specific gases, the response was combined into 4-dimensional (4D) matrix (according to the number of sensor groups), which was projected onto a lower dimensional space using unsupervised principal component analysis (PCA) and local linear embedding (LLE), as well as supervised linear discriminant analysis (LDA) machine learning algorithms. Figures 4a–c show corresponding 2D plots after the dimensionality reduction for four studied gases at varied concentrations in dry air, while the data used can be found in Supplementary Fig. S7 . On the PCA plot the regions corresponding to NO_2_, NH_3_ and Et-SH do not overlap at all leading to clear discrimination between these gases in the dry air. H_2_S region overlaps with NH_3_ at low concentrations close to LOD and then approaches Et-SH area at higher concentrations forming a region corresponding to thiol-containing gases often associated with spoiled food and used as natural gas odorants. LLE provides similar results, but also features with linear directions along which the concentration for each of the gases increases leading to not only qualitative, but also quantitative gases recognition. LDA separates each of the gases even better than the other methods investigated clearly defining the corresponding regions above 80 ppb concentration (except 40 ppb of Et-SH, which arise in H_2_S region).

Figure 4d–f show PCA, LLE and LDA 2D plots for the measurements in the humid air at 95% RH with the source data presented in Supplementary Fig. S8  . Water vapor somewhat complicates the gases recognition with PCA and LDA methods, since H_2_S region overlaps much stronger with NH_3_ region due to higher LOD resulting in a more difficult discrimination between them, while NO_2_ is still fully distinguishable from the rest at the concentrations as low as 100 ppb. Moreover, Et-SH region fully separates from NH_3_ at concentrations above 120 ppb. LDA plot demonstrates full separation of Et-SH and H_2_S, while both still overlap with NH_3_. Thus, the electronic nose array with a proper pattern recognition algorithm is suitable for detection and discrimination of nitrogen dioxide, ammonia and thiols in dry air, while facing more difficulties at 95% RH.

Further improvements can be achieved if focusing on the reducing gases alone, which is shown in Supplementary Fig. S10 . In the dry air both PCA and LLE feature linear projection of the concentration growth, while still struggling to distinguish analytes at 95% RH. However, clear separation between all three reducing gases was achieved for both dry and humid air using LDA pointing out a great potential for the gases recognition using supervised techniques and appropriate training dataset.

### Meat spoilage detection

**Figure 5:**
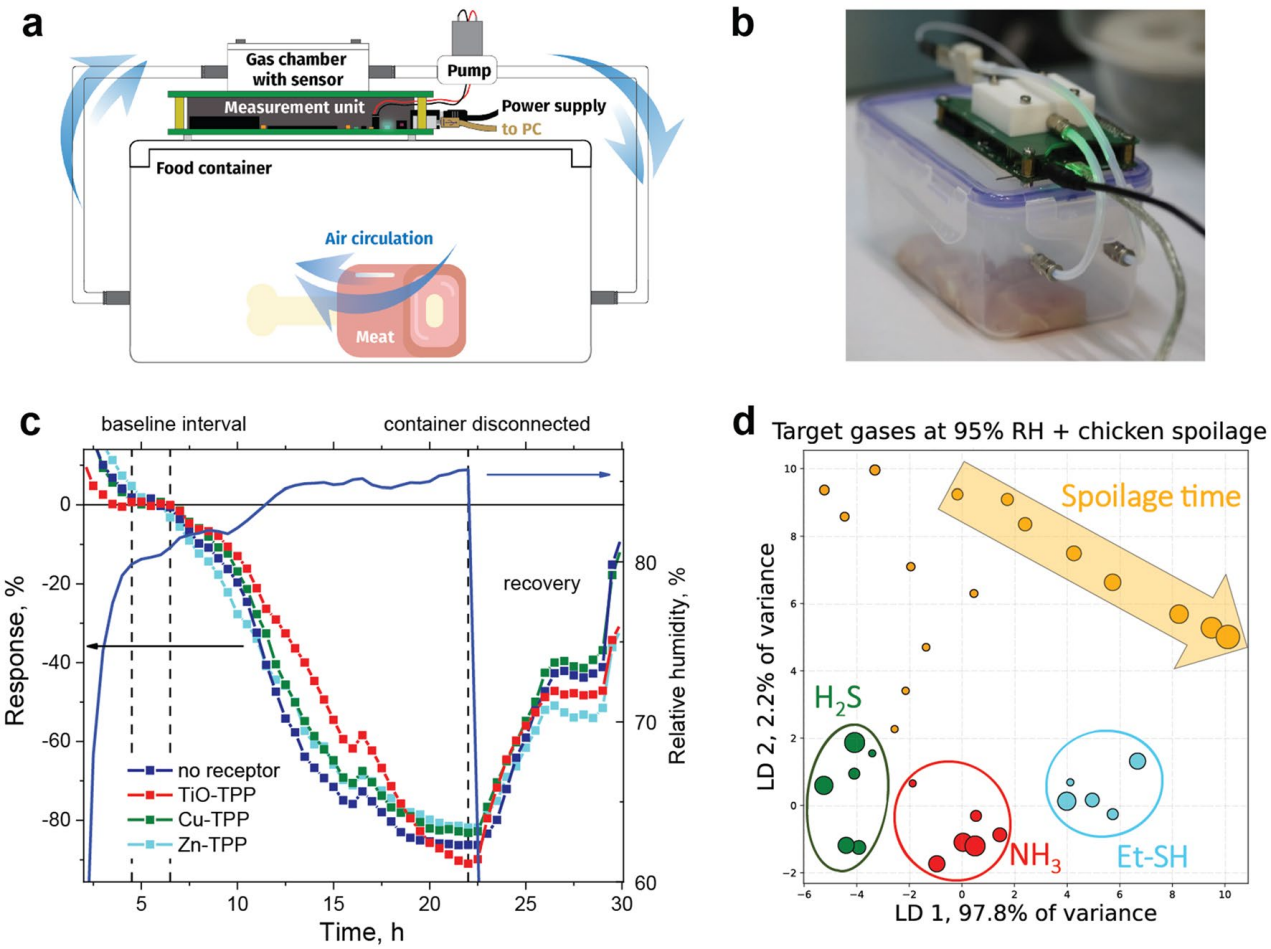
Food experiments. (**a**) Scheme of the meat spoilage experiment. (**b**) Setup photo with a piece of chicken breast, the electronic nose and a membrane micropump mounted on the top. (**c**) Response-time dependence over spoiling meat for the sensor groups with different receptor layers, relative humidity measured with a reference sensor is plotted in blue line and linked to the right axis. (**d**) LDA diagram of the signal growth during meat decomposition with response measurements of the target analytes considered as a training dataset and the data recorded during meat spoilage as a test dataset, each dot corresponds to 1 h interval and dot size with the arrow—to its growth direction.

An automated control of food freshness by gas sensors requires resistivity to humidity and sensitivity towards volatile thiol-containing compounds, which are related to proteins decomposition at 100 ppb range^5,7,59^. Fast, sensitive and reusable sensors are long awaited in food industry to replace long and expensive bacteriological methods^4,60^. Recent work on inorganic selective gas sensors array appeared to be capable for a particular food decomposition assessment^7^, although protein decomposition has a complex profile that is difficult to be linked to a single responsible marker such as hydrogen sulfide or ammonia. Moreover, storage of multiple foods together blurs the signal with interfering volatiles. To demonstrate discriminative ability and stable operation in the air with high relative humidity of the electronic nose presented in this work on a practical problem, we have studied gases released during chicken breast spoilage. Figure 5a,b shows the experiment scheme used and setup picture. The device was connected to a food container filled with a piece of meat through short PTFE tubes and a membrane micropump. Figure 5c shows the signal evolution over 30 h with initially fresh chicken and slowly growing relative humidity. On the 4th hour the humidity approached its saturated value leading to the sensor signals stable plateau with a duration of 2 h, which values have been used as a baseline for the sensor responses normalization. Then the response amplitude starts to grow during the next 15 h approaching 90% on the 21st hour of the meat spoilage corresponding to appearance of some new analytes. Substantial response value of 30% is achieved by the 10th hour of the storage, which can be considered as a threshold value between the fresh and spoiled product**.** The reference measurements over the container filled with pure water (see Supplementary Fig. S11 . ) indicate that this growth is related neither to bare humidity oscillations nor to the sensor degradation during the experiment timescale. After the meat was spoiled, the container was disconnected from the electronic nose and pure air was allowed to enter the gas chamber by means of the micropump. This have led to the sensor array recovering proving the whole system reusability.

The signal variations between the sensor groups with different receptors allowing catching the odor “fingerprint”. Multidimensional response corresponding to different receptor layers over the spoiling meat considered as a test dataset were analyzed with machine learning methods mentioned above. Since oxidative gases such as NO_2_ are not expected to be emitted during meat spoilage, only the reducing analytes (NH_3_, H_2_S, Et-SH) obtained in the humid air (presented in Supplementary Fig. S10) were considered as a training dataset. In Figure 5d one can see that the meat spoilage measurements projected onto a linear discriminants space initially lies between NH_3_ and H_2_S region then moving to unmarked area of the plot. However, the saturated signal is closer to Et-SH region among any of the studied analytes in terms of Euclidian distance, which allows speculating that there are mostly thiol-containing volatile decomposition products that is consistent with the other related studies^4,5^. The other dimensionality reduction plots of the measurements over spoiling chicken with training dataset comprised from either only reducing or all the gases studied can be found in Supplementary Fig. S13. All the 2D projections demonstrate that spoilage gases initially lies close to NH_3_ and H_2_S then moving to unmarked region. On the LDA plots in Fig. 5d and in Supplementary Fig. S13f with the measurements are still is the closest to Et-SH region.

Thus, we have demonstrated the potential of application of the presented electronic nose to comparison of the unknown gas samples with a training dataset tested before. With more training on various simple gases and food products, the system could evaluate the composition or even type of a food spoiled, which can find application in smart fridges, where many products are usually stored together. In general, this is not limited to four simple gases presented, but also applicable to automated quality OK/not OK test for food or cosmetics production.

## Conclusions and outline

In this work a versatile platform for creation of fully integrated OFET-based cross-selective gas sensors array and portable device providing its simultaneous measurements was developed. Each of the sensors based on different ultrathin active layers, is a reproducible reusable device itself with a stable baseline capable to detect toxic gases such as ammonia, hydrogen sulfide, nitrogen dioxide or ethanethiol with the LODs in 30–470 ppb range. Combined with unsupervised machine learning algorithms of PCA this electronic nose is capable not only to detect these toxic gases in the ppb concentration range, but also to discriminate them efficiently in a wide relative humidity range up to 95% while LLE even provide potential for quantitative determination. Supervised LDA approach leads to better discrimination of each gases, that even such chemically similar gases as hydrogen sulfide and ethanethiol can be distinguished. This highlight potential of the electronic nose to be pretrained for desired tasks. A large number of sensors within the array and its averaging increase the measurements reliability. The approach elaborated could be used for stand-alone low cost IoT air quality sensors suitable for real-time environmental monitoring with the specific polutants discrimination. Furthermore, its great potential for food spoilage determination was demonstrated on a chicken breast with the ability to see clearly its decay just after 10 h of storage at room temperature due to reducing gases emission. Moreover, we show potential for spoilage gases composition determination based on training dataset which however have to be widen. The electronic nose reported is promising for the food decomposition assesments after appropriate training as compared to selective sensors, while it costs much less due to solution processing used for device production, sensors reusability and simple circuitry. Further system integration with hardwired machine learning or neuromorphic processors would provide even higher computation efficiency and cost reduction^61,62^. The platform elaborated is not limited to four toxic gases reported and pave the way for the development of advanced devices integrated into smart fridges or storage rooms for selective food spoilage detection with the other products in the background. Moreover, such approach could be further extended towards an exhaled breath analysis due to outstanding sensitivity to nitrogen dioxide in the humid air.

## Methods

### Materials

The synthesis of organic semiconductor, which was organosilicon derivative of non-symmetric dialkyl-benzothienobenzothiophene (D2-Und-BTBT-Hex)–1,3-*bis{*11-(7-undecyl[1]benzothieno[3,2-b][1]benzothio-2-yl)hexyl}-1,1,3,3-tetramethyldisiloxane was performed as described elsewhere^33^_._ Synthesis of 5,10,15,20-tetraphenylporphyrin of titanyl (TiO-TPP); 5,10,15,20-tetraphenylporphyrin of copper (Cu-TPP) and 5,10,15,20-tetraphenylporphyrin of zinc (Zn-TPP) were performed as described elsewhere^63,64^. Organic solvents (toluene, ethanol) were purchased from Acros Organics, Polymethyl-methacrylate (PMMA) M_w_ = 230 000 was purchased from Sigma-Aldrich and used as received. Ultrapure deionized water (18MΩ) was made by D-301 deionizer (Akvilon, Russia).

### Sensor array fabrication

Doped silicon substrates with a 200 nm of thermally grown oxide were used as a substrate. The standard procedure for substrates surface cleaning was carried out with two-stage ultrasonication in acetone and isopropyl alcohol. After that the substrates were washed with ultrapure deionized water followed by drying in nitrogen flow. Clean silicon oxide surface was treated with oxygen plasma and covered with spin-coated PMMA buffer layer to reduce surface charge trap density. The solution was prepared by dissolving PMMA in toluene at concentration of 10 g L^−1^. PMMA deposition on silicon surface was performed by spin-coating at the substrate rotation speed of 2000 rpm for 90 s and then dried at vacuum oven at 110 °C for 2 h. This resulted in 50 nm layer with 0.26 nm RMS roughness addressed with Ntegra Prima II atomic force microscope and ETALON HA_FM 77 kHz probes (NT-MDT, Russia). Gold source and drain electrodes were thermally evaporated in vacuum through shadow masks forming 20 pairs of source-drain electrodes with 30 and 1000 um channel width and length, respectively. These steps are depicted in Supplementary Fig. S1. Capacitance per area of 14.5 ± 0.4 nF/cm^2^ was measured with E7-20 immittance-meter (MNIPI, Belarus).

To fabricate cross-selective sensor array a single substrate was firstly covered with a monolayer of D2-Hept-BTBT-Hex organic semiconductor Langmuir-Shaeffer (LS) film forming 20 OFETs. Widely used spin-coating of OSC onto a polymer dielectric is able to dissolve the latter and increase its roughness if orthogonal solvent is not used, while Langmuir technique allows to remove organic solvents prior to the film deposition stage and preserves a smooth PMMA surface. After that the substrate was partially modified by 3 different metalloporphyrin Langmuir–Blodgett (LB) receptor layers successively deposited from diverse sides of the substrate as shown in Supplementary Fig. S2. This resulted to the same substrate consisting of 6 bare sensors, 4 sensors covered with TiO-TPP, 3—with Cu-TPP and 3—with Zn-TPP receptor layers; top view of the substrate is shown in Fig. 3. The LB semiconductor and LS receptor films deposition processes are described in details elsewhere^39^. The 4 corner sensors covered with double receptor layers were also sensitive to the studied analytes but were excluded from the consideration to isolate pure receptor effect.

### OFET properties

The non-modified OFETs show average saturated hole mobility of 0.09 ± 0.03 cm^2^/Vs and threshold voltage of 4.3 ± 0.6 V as an average of 40 transistors with reliability factor r = 67%^65^. A typical transfer curves of a single device and its degradation over 1 year at ambient air shown in Supplementary Fig. S3 were measured using 2634B 2-channel source-meter (Keithley, USA) and probe station 100 (Printeltech, Russia). They operate for more than one year while stored at normal conditions due oxidative and thermal stability of BTBT-derivatives. Shift of the transfer curve while storage at ambient conditions have occurred due to increased threshold voltage and the saturated current have decreased by 60% to 500 nA, which is still remains 2 orders of magnitude higher than the device noise level.

### Electronic circuit design and signal measurements

All the sensors were fully integrated into a portable device in order to demonstrate its capabilities and obtain the whole array sensor response simultaneously. The device, whose principal scheme and photos are shown in Fig. 1f–h, is a compact multi-channel source-meter. The source part consists of two inverting high-voltage operational amplifiers, supplied from a double-channel DC–DC converter, and digital-to-analog converters of the microcontroller (MCU). This allows applying voltages of desired magnitude and polarity independently to the gate and draining electrodes of the OFET array. The measurement of OFETs channel current is performed via a I-to-V conversion using another transimpedance amplifier. This circuit creates a voltage drop proportional to the input current on the feedback resistor, which is then measured by precision sigma-delta analog-to-digital converters (ADC). The MCU used is incorporated with 3 sigma-delta ADCs with analog multiplexers, which allows measuring 21 channels in total. Pseudo-simultaneous measurements were achieved via a quick scan with the integrated multiplexer. Since the sigma-delta ADC used tends to have gain and offset errors, an external calibration was performed. A linearly rising set of voltages was applied to the ADC input by using E3631A (Hewlett-Packard, USA) laboratory power supply. The actual amplifier gain and offset were calculated based on the linear dependence of the voltage measured with the ADC versus the applied one. Since the current-to-voltage converter’s gain and the noise performance are defined by the feedback resistor, thus precise metal-film resistors were used, the actual values of which were measured with 34410A (Agilent, USA) multimeter. A compact and power efficient design was achieved by utilizing the peripherals set of the microcontroller in full. The use of self-calibrating zero-drift operational amplifiers allows minimizing the offset drift during long-time measurements. The low voltage power supply is based on a power management integrated circuit (IC), which contains two step-down DC-DC converters, a Li-ion battery charger and a power source switch circuitry. The device is powered by 5 V delivered by either integrated rechargeable 300 mAh Li-ion battery, wall adapter or USB cable, power consumption during the measurements is as low as 400 mW. The amplifiers and analog part of the MCU are powered by a low-noise linear regulator. During the sensor response measurements fixed voltages V_sd_ = V_sg_ =− 30 V were used at a duty cycle of 0.1% to perform pulsed measurements with low sensor baseline drift. Further signal conditioning included digital low pass filter running on the MCU to reduce noise level. The measurements results were then sent to a PC, where they were further processed via accompanying software written on C#.

Custom designed gas chamber incorporates an array of pogo pins for substrate connection, a commercial humidity and temperature sensor, and a thin resistive heater with a thermistor feedback, touching the substrate, which allows operation even at low temperature. The PID-control running on the MCU allows to maintain the temperature of the substrate slightly above the ambient to avoid water condensation while using at low temperatures, substrate temperature during experiments were set to 30 °C. Total device dimensions do not exceed 60 × 90 × 30 mm.

### Sensors characterization and data analysis

Gas mixtures were prepared using GGS-K mixing station (Monitoring, Russia) with calibrated permeation tubes (Analitpribor, Russia and Monitoring, Russia) as an analyte source. Dry (RH < 0.1%) clean air was obtained from zero air generator (Khimelectronica, Russia) and was used as a carrier gas diluting permeable analyte flow in a predefined ratio, flow rate was kept at 0.8–1 L min^−1^. The station was connected to a PC with the software for experiment automation. Since ethanethiol has much lower threshold limit values, the tube with lower permeation rate was used determining the lower available concentration range of 40–200 ppb. NO_2_ was studied in 100–1000 ppb range, while NH_3_ and H_2_S were studied in the 100–1500 ppb range.

A Dreschel bottle with distilled water was used for humid air composition. Resulting humidity was controlled by IVTM-1 (Eksis, Russia) hygrometer and compact SHT25 (Sensirion, Switzerland) temperature and relative humidity (T/RH) sensor integrated into the electronic nose gas chamber. Volatile organic compounds (VOCs) vapor was also delivered from the Dreschel bottle filled with a purified liquid whose concentration were calculated based on a saturated vapor value and dilution ratio. CO_2_ was delivered from a calibrated cylinder.

Each sensor response measurement with a specific gas included 10 min long exposition at each concentration followed by 50 min recovery in clean air flow kept at humidity of the experiment. The response value is given by the current normalized to the baseline value picked at clean air with either zero or some specific relative humidity of the experiment. Each sensor response is presented by the average value in the interval from 7 to 10th minutes of the gas exposure, which were then averaged over a group of identical sensors on the substrate and plotted on the response-concentration curves with its standard error. The interval was chosen considering the average t_90%_ time of 490 ± 90 s. Positive response values corresponded to oxidative gases such as NO_2_, while negative—to reducing gases such as ammonia and thiol-containing compounds studied. A limit of detection (LOD) was estimated as a concentration corresponding to the device response value that is three times higher than the baseline noise level^66^. A sensitivity was estimated as a slope of linear fit of the response-concentration dependence. Calculated sensitivities and LODs for all studied gases and each sensor group are summarized in Supplementary Table S2 .

Averaged response concentration dependence related to a specific subset of the sensors were compiled to a response matrix based on which further data analysis were performed. The matrix was scaled to unit variance that refers to mean centering followed by dividing by the standard deviation prior to two-dimensional (2D) principal component analysis (PCA), Local Linear Embedding (LLE) and Linear Discriminant Analysis (LDA) for data clustering visualization. Scaling and dimensionality reduction were done using scikit-learn Python library. Dot size on the diagram was used to represent either the concentration growth in the case of the target analytes (not in scale) study or the increase of spoilage time in the case of the experiments with a meat. Explained variance related to PCA and LDA is given on the corresponding axis title. LLE was used with neighbors amount k = 6.

### Food experiments

Experiments with food were performed with the sensor chamber connected to a sealed food container of 0.6 L volume through short PTFE tubes and a membrane micropump inducing air flow in a closed circle (Fig. 5a). Fresh 50 g piece of a chicken breast bought in the morning at the local organic food supplier were placed into the container and stored at room temperature during which the sensor array signal was measured each 2 min. Humidity slowly produced by a piece of meat were measured with T/RH sensor integrated into the electronic nose gas chamber. Its value has grown to 90% of the saturated relative humidity value in 4 h after the container was connected leading to the sensor signals stable plateau with duration of 2 h, which mean values have been used as a baseline for the sensor responses normalization. The response was averaged between groups of the sensors in the array with different receptors layers. The experiment has lasted until the substantial signal growth of the response were obtained and the response was measured each 2 min. To analyze the composition of gaseous spoilage products the signal was reduced to 1-h long intervals, which was then projected onto a 2D principal component space altogether with the target gases at humid air considered as a training dataset. Comparison between classes on 2D plots were done using Euclidean distance. A piece of meat at the beginning of the experiment and after 24 h of storage at room temperature is presented in Supplementary Fig. S12 **.** To recover the sensor array after the experiment, the container was disconnected and room air flow was induced through the sensor chamber via the membrane micropump which resulted in full recovery. As a referencing experiment the sensor array response was recorded above another container filled with 100 g of distilled deionized water kept at room temperature to mimic slow relative humidity saturation in the volume inside the container. Mean response of the group of six non-modified sensors to water vapor over 30 h in Supplementary Fig. S11  shows a lack of the significant response growth related to the sensor degradation or pure humidity related effects and referring the response growth to appearance of new volatile compounds during the meat spoilage.

Online Content Methods along with any additional Extended Data display items and tables are available in the online version of the paper; references unique to these sections appear only in the online paper.

## Supplementary Information

Supplementary Information

## Data Availability

The data that support the findings of this study are available from the corresponding author upon reasonable request.
