# Supplementary material for: Fully integrated ultra-sensitive electronic nose based on organic field-effect transistors
Source: Sci Rep. 2021 May 21;11:10683. doi: 10.1038/s41598-021-88569-x (PMC8140082; doi:10.1038/s41598-021-88569-x)
Supplement: Supplementary file 1 — Supplementary Information [file 41598_2021_88569_MOESM1_ESM.pdf]

Supplementary information

Versatile platform for ultra-sensitive electronic nose based on organic field effect transistors

Daniil S. Anisimov[1,2], Victoria P. Chekusova[1,2], Askold A. Trul[1,2], Anton A. Abramov[1], Oleg V. Borshchev[1], Elena V. Agina[1] and Sergey A. Ponomarenko[1*]

[1] Enikolopov Institute of Synthetic Polymeric Materials of the Russian Academy of Sciences, Profsoyuznaya str. 70, 117393, Moscow, Russian Federation

[2] Printeltech LLC, Profsoyuznaya str. 70, 117393 Moscow, Russian Federation

* E-mail: ponomarenko@ispm.ru

Supplementary discussion

The OFET-based cross-selective gas sensors arrays reported in the literature up to now are presented in Supplementary Table 1. Most of them are only able to distinguish analytes at 100 – 1000 ppm range and operate in vacuum, nitrogen or dry air failing to face humidity. Moreover, all of them were in fact single sensors measured one by one with laboratory equipment that does not allow operation as electronic nose just virtually.

This work reports on the first real OFET-based electronic nose. It is able to detect and distinguish NO_2, NH_3, H_2S and Et-HS qualitatively at ppb-level (down to 0.04 ppm) in the air with varied humidity range up to 95%. The presented approach allows placing the whole sensors array on a single substrate by solution-based Langmuir technique. The sensor array is fully integrated into a portable device able to simultaneously measure responses in multichannel mode and is suitable for manifold of practical applications such as environmental monitoring, food industry and exhaled breath analysis.

Supplementary Table S1 | OFET-based cross-selective gas sensors arrays reported in the literature

Materials	Distinguishable analytes	Lowest measured concentration, ppm	Carrier gas	Device presented	Reference
6T-derivatives, phthalocyanines	alcohols, ketones, thiols, nitriles, esters, and aromatic compounds *	10-100	Dry air/N_2	No	1
pentacene, P3HT, P3OT	milk/water vapor	–	Dry N_2	No	2
P3HT-derivatives	Alcohols, acids, bases, carboxylic acids, and aldehydes *	10-50	Dry air	No	3
polytriarylamines	DMMP, acetone, methanol and propanol	2500 -500 000	Dry N_2	Yes**	4
polycyclic aromatic hydrocarbons	alcohols, alkanes, and aromatic compounds	10-8000	Air, RH 0-40%	No	5
8-NTCDI, DMP-NTCDI, CuPc	Acetic acid, acetone, IPAM, water, DMMP, H_2O_2, hexane and toluene ****	1500 -11 000	Vacuum	No	6
PDPPHD-T3***	o,m,p-xylene isomers	40-320	Dry N_2	No	7
Diketopyrrolopyrrole copolymers	hexane, octane, decane, benzene, toluene, p-xylene	700-7000	Dry air	No	8
**D2-Und-BTBT-Hex + metalloporphyrins**	**NO_2, NH_3, H_2S, Et-HS**	**0.04-0.5**	**Air, RH 0-95%**	**Yes**	**This work**

* only qualitative results are presented
** non portable device, which was used for measurements of single OFETs in series
*** single ambipolar OFET is considered as 6 virtual sensors due to multiparametric detection
**** only aromatic / non-aromatic and polar / not-polar VOC classes are distinguished

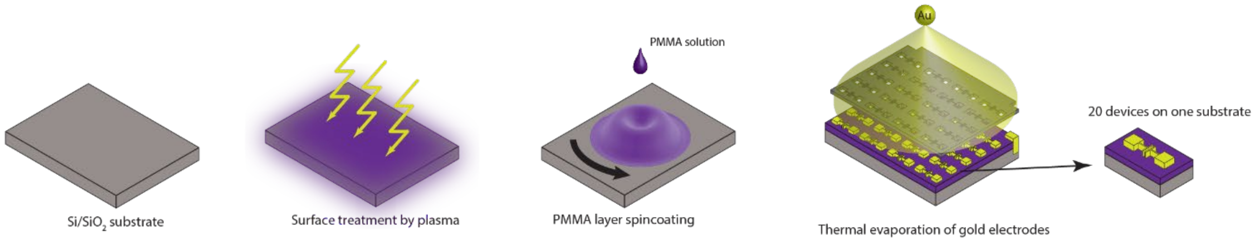

Figure S1 | Illustration of substrate preparation process

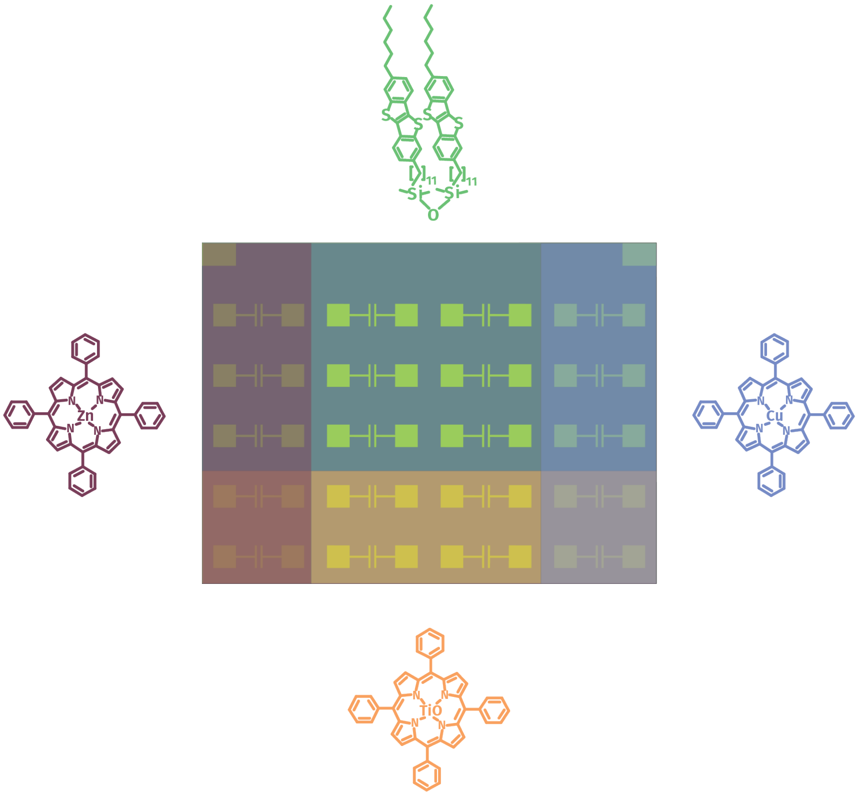

Figure S2 | Sensor array layout. Top view on the array of 20 sensors on a single substrate partially modified by different receptors with its chemical structure.

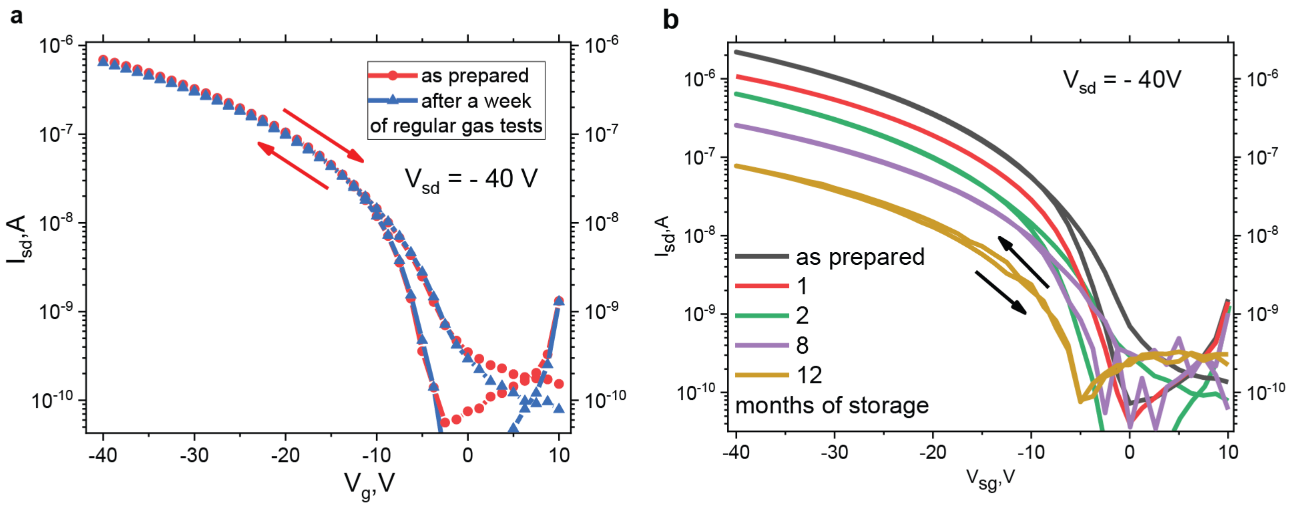

Figure S3 | Stability measurements of the monolayer OFET without a receptor layer. **a**, on a short term over one week and **b**, on a long term over one year of storage under ambient conditions, while regular gas sensing experiments were performed.

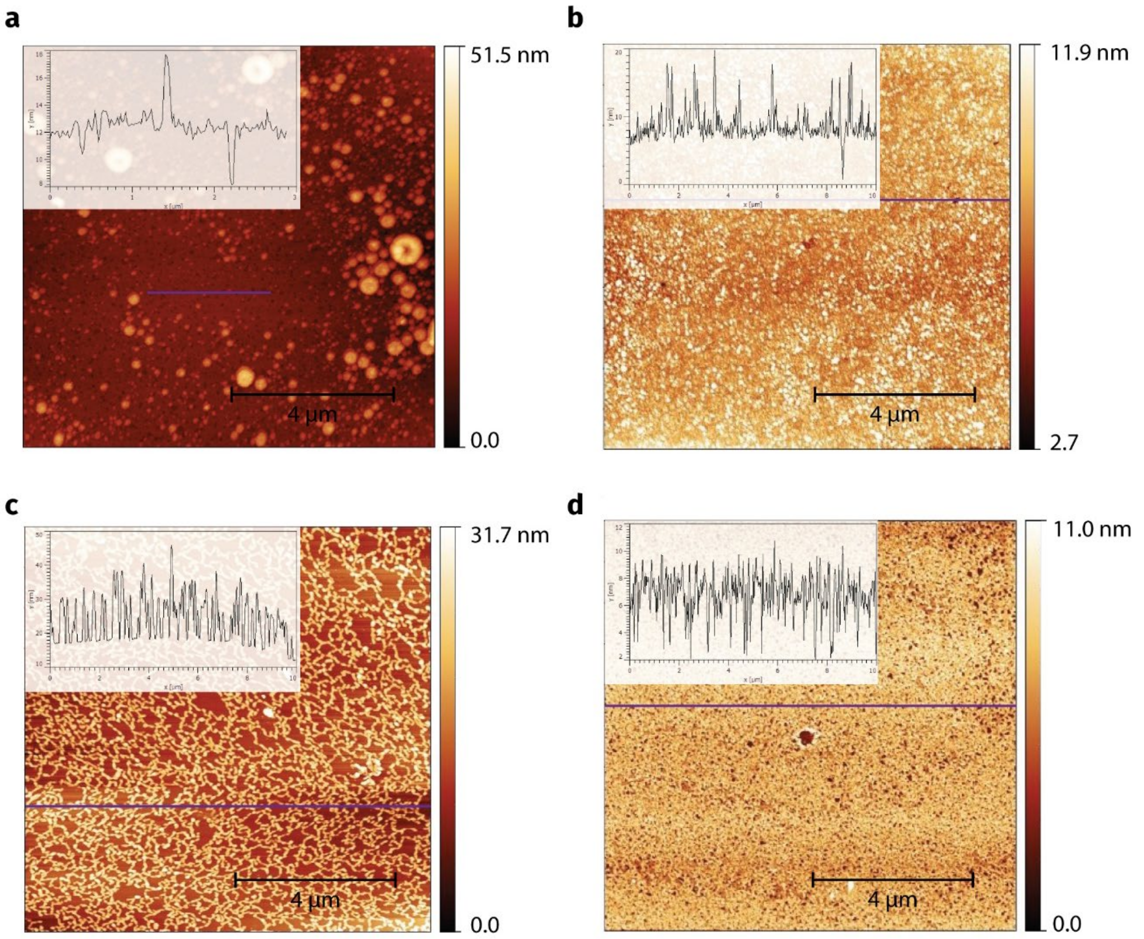

Figure S4 | Atomic force microscopy images of the films studied. a, D2-Und-BTBT-Hex. **b**, TiO-TPP, **c**, Cu-TPP and **d**, Zn-TPP receptor layers.

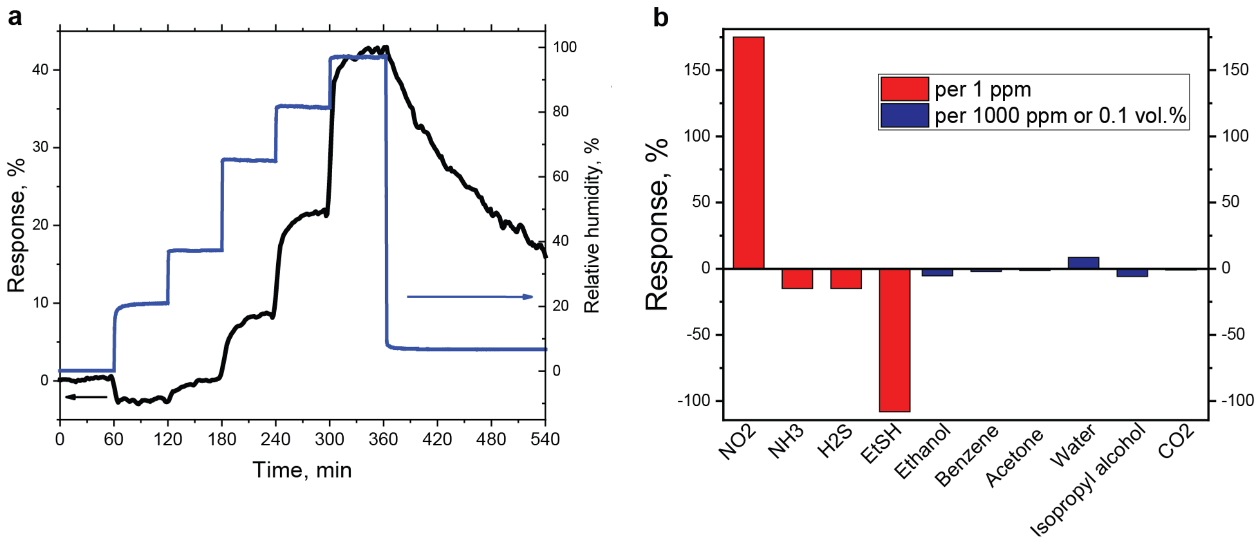

Figure S5 | Non-modified sensors cross-sensitivity. a, Mean response of 14 sensors without receptor layers to different relative humidity. **b**, cross-selectivity diagram of non-modified sensors to target analytes and VOCs.

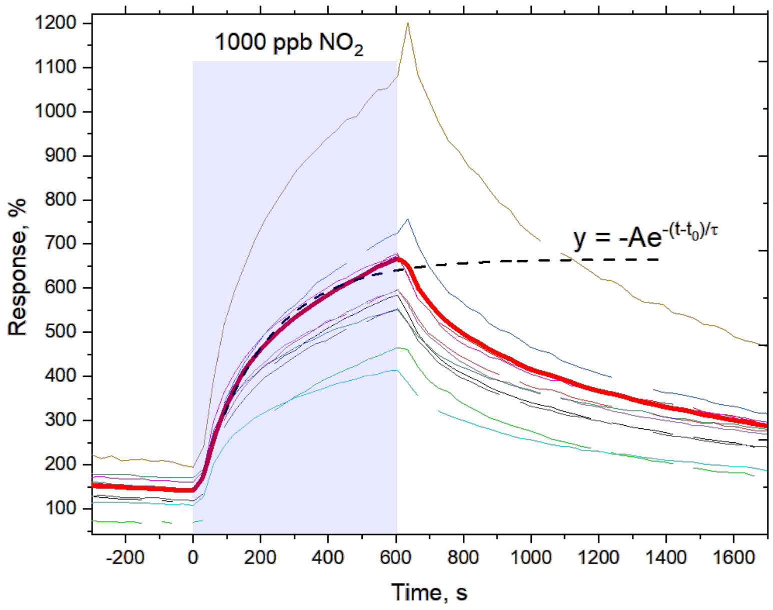

Figure S6 | Sensor response to NO_2 reproducibility measurements. Response-time (left axis) dependence of 6 sensors without receptor layers to a single exposure of 1000 ppb of NO_2 in humid air at 95% RH compiled from 2 repeated experiments (color lines) and its mean value (bold red line) and exponential approximation (dotted line) with NO_2 pulse shown in blue.

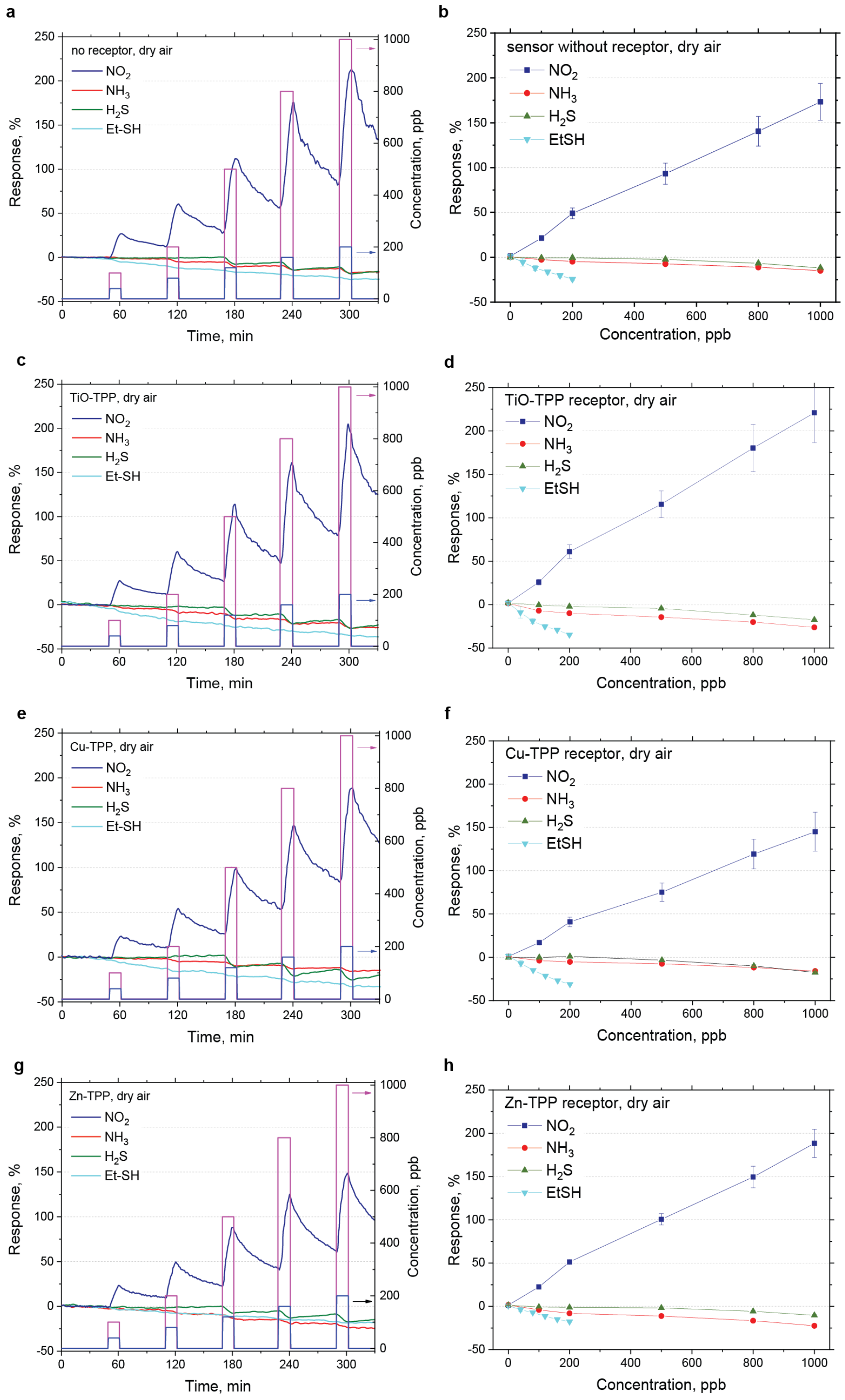

Figure S7 | Response of the sensors with different receptor layers to target analytes in dry air. Response-time dependence is shown in the left column and corresponding response-concentration dependence in the right column. **a**,**b**, Sensors without a receptor layer, **c**,**d**, with TiO-TPP, **e**,**f**, Cu-TPP and **g**,**h**, Zn-TPP receptor layers. Corresponding pulse concentration is shown on the right axis in magenta for NO_2, NH_3, H_2S and in blue for Et-SH. Mean response among the identical sensors of the array is presented with its standard error.

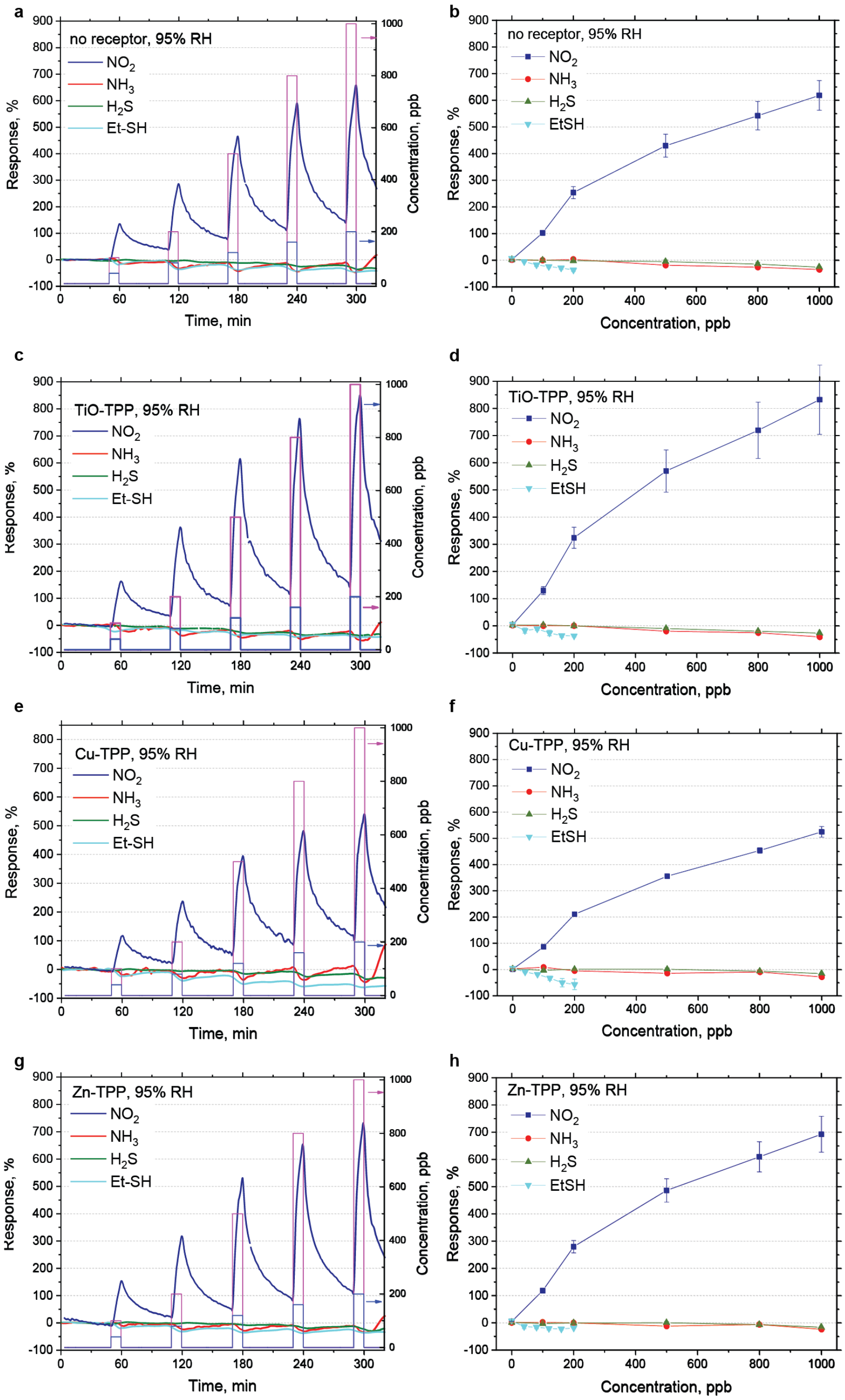

Figure S8 | Response of the sensors with different receptor layers to target analytes in humid air at 95 % RH. Response-time dependence is shown in the left column and corresponding response-concentration dependence in the right column. **a**,**b**, Sensors without a receptor layer, **c**,**d**, with TiO-TPP, **e**,**f**, Cu-TPP and **g**,**h**, Zn-TPP receptor layers. Corresponding pulse concentration is shown on the right axis in magenta for NO_2, NH_3, H_2S and in blue for Et-SH. Mean response among the identical sensors of the array is presented with its standard error.

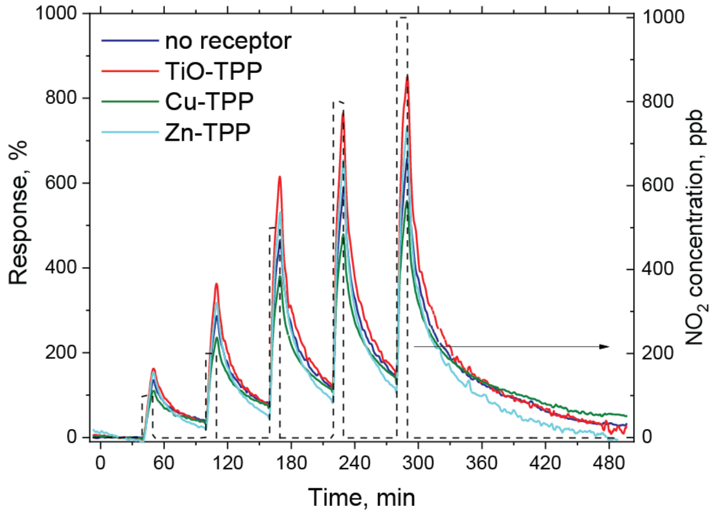

Figure S9 | Mean response of the sensor groups with different receptor layers: TiO-, Cu-, Zn-TPP and the sensors without a receptor layer to NO_2 pulses at 100, 200, 500, 800 and 1000 ppb concentrations in humid air at 95% RH.

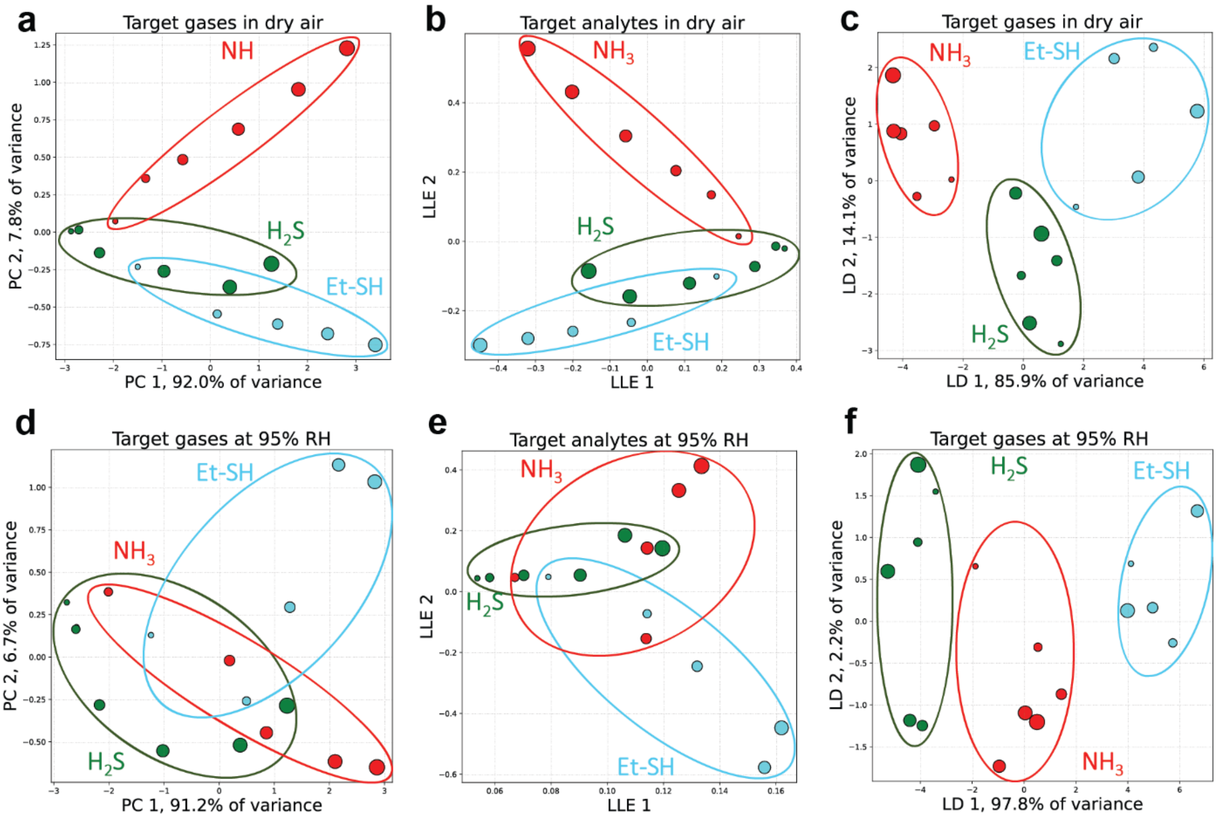

Figure S10 | Dimensionality reduction plots related to reducing gases studied in dry air (top row) and at 95% RH (bottom row) a,d, Principal Components Analysis (PCA). **b,e,** Local Linear Embedding (LLE). **c,f,** Linear Discriminant Analysis (LDA).

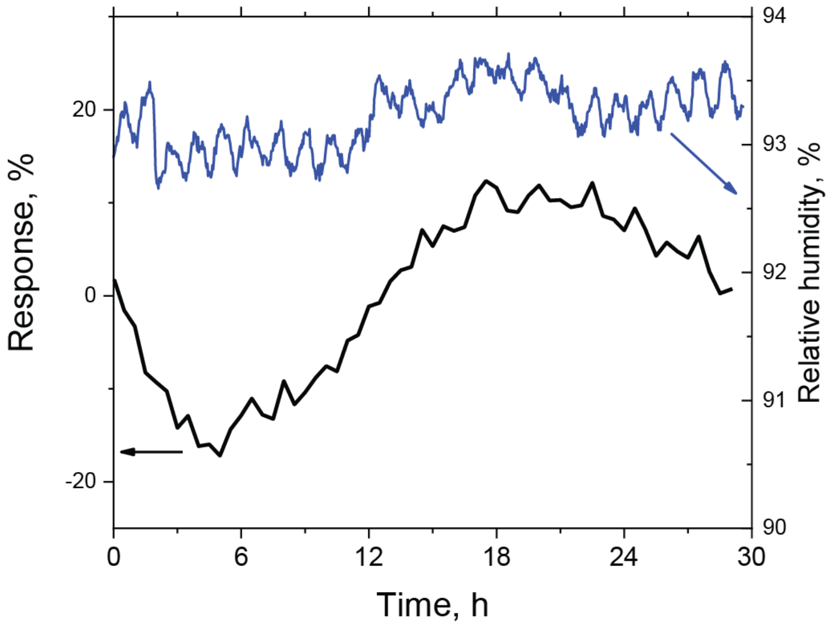

Figure S11 | Reference measurement of 6 non-modified sensors of the array over a food container filled with pure water. There are fluctuations around zero of ± 12% over 30 hours of the measurements with no further growth as compared to the experiment with a piece of meat.

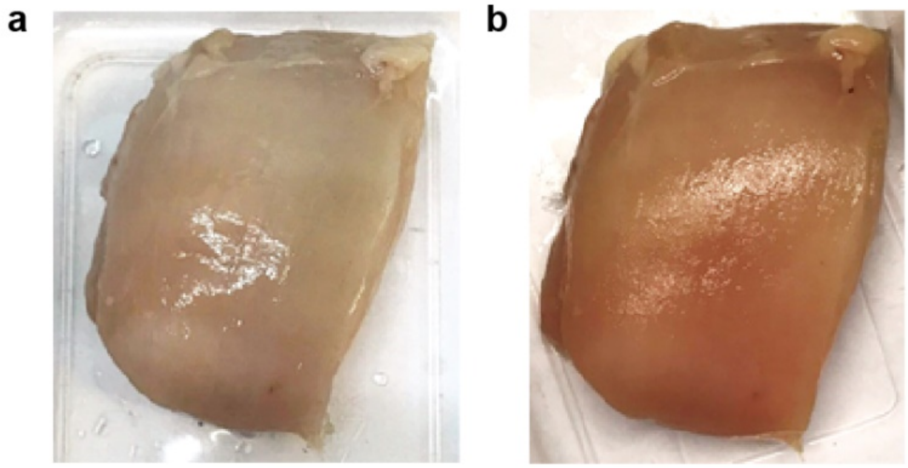

Figure S12 | A piece of chicken at the beginning of the experiment as purchased (**a**) and after 24 hours of storage at room temperature (**b**). The meat slightly darkens when spoiled.

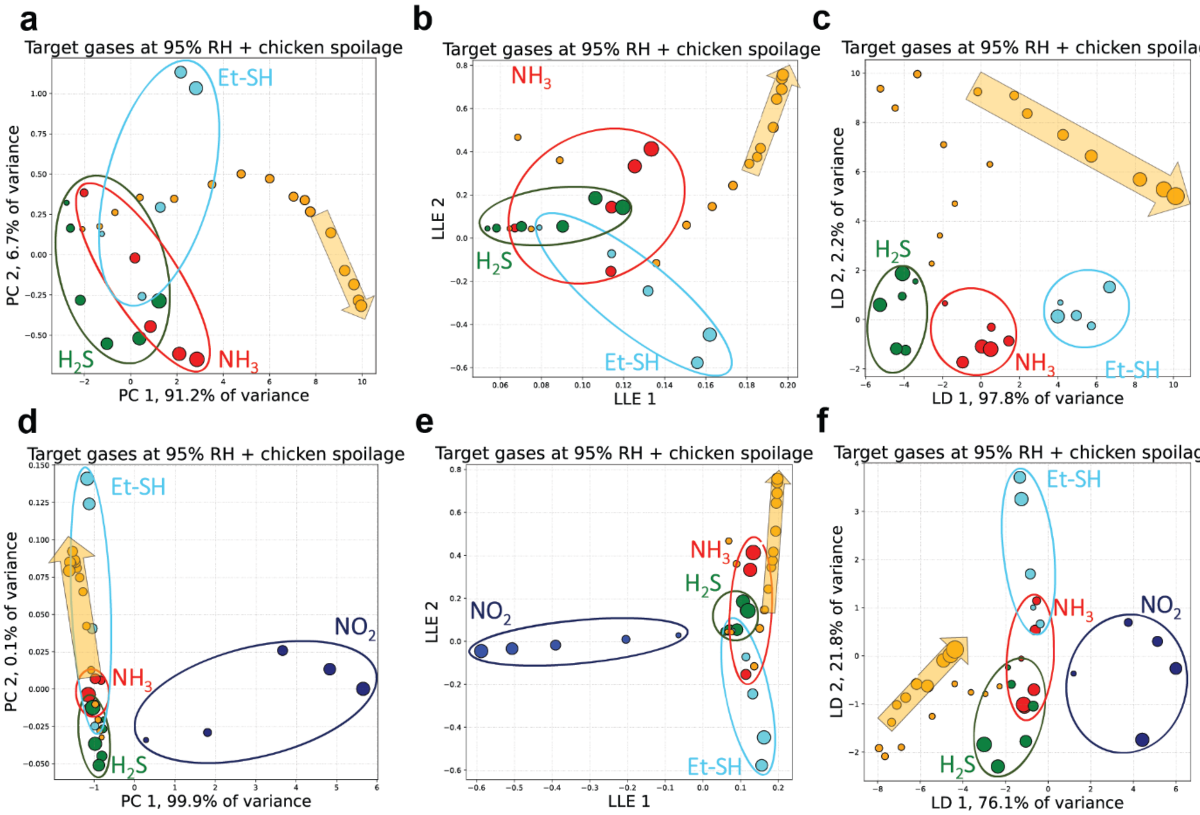

Figure S13 | Dimensionality reduction plots related to the reducing gases studied in dry air (top row), at 95% RH (middle row) and with chicken spoilage measurements projected on the correspondint coordinates (bottom row). a,d, Principal Components Analysis (PCA). **b,e,** Local Linear Embedding (LLE). **c,f,** Linear Discriminant Analysis (LDA).

Supplementary Table S2 | Limit of detection and sensitivity of the sensors with different receptor layers to all studied analytes in dry air and in humid air at 95% RH.

Receptor	Gas	RH, %	Sensitivity, % per ppm	LOD, ppb
no receptor	NO_2	0	175±14	40
	NO_2	95	472±35	30
	NH_3	0	-16±2	120
	NH_3	95	-46±8	250
	H_2S	0	-19±3	470
	H_2S	95	-43±6	290
	Et-SH	0	-114±6	30
	Et-SH	95	-196±22	40
TiO-TPP	NO_2	0	227±20	30
	NO_2	95	665±53	20
	NH_3	0	-19±2	350
	NH_3	95	-46±5	170
	H_2S	0	-26±1	390
	H_2S	95	-34±5	320
	Et-SH	0	-135±9	70
	Et-SH	95	-207±48	20
Cu-TPP	NO_2	0	149±14	30
	NO_2	95	425±28	40
	NH_3	0	-13±2	280
	NH_3	95	-24±6	640
	H_2S	0	-28±4	450
	H_2S	95	-37±6	710
	Et-SH	0	-133±6	50
	Et-SH	95	-352±24	30
Zn-TPP	NO_2	0	167±3	30
	NO_2	95	545±48	40
	NH_3	0	-16±2	180
	NH_3	95	-27±4	400
	H_2S	0	-17±3	490
	H_2S	95	-36±7	590
	Et-SH	0	-88±5	20
	Et-SH	95	-90±17	20

References

1 Crone, B. *et al.* Electronic sensing of vapors with organic transistors. *Applied Physics Letters* **78**, 2229-2231, doi:10.1063/1.1360785 (2001).

2 Liao, F., Chen, C. & Subramanian, V. Organic TFTs as gas sensors for electronic nose applications. *Sensors and Actuators B: Chemical* **107**, 849-855, doi:10.1016/j.snb.2004.12.026 (2005).

3 Chang, J. B. *et al.* Printable polythiophene gas sensor array for low-cost electronic noses. *Journal of Applied Physics* **100**, 7, doi:10.1063/1.2208743 (2006).

4 Wedge, D. C. *et al.* Real-time vapour sensing using an OFET-based electronic nose and genetic programming. *Sensors and Actuators B: Chemical* **143**, 365-372, doi:https://doi.org/10.1016/j.snb.2009.09.030 (2009).

5 Bayn, A., Feng, X., Müllen, K. & Haick, H. Field Effect Transistors Based on Polycyclic Aromatic Hydrocarbons for the Detection and Classification of Volatile Organic Compounds. *ACS applied materials & interfaces* **5**, 3431-3440, doi:10.1021/am4005144 (2013).

6 Huang, W. *et al.* Diverse Organic Field-Effect Transistor Sensor Responses from Two Functionalized Naphthalenetetracarboxylic Diimides and Copper Phthalocyanine Semiconductors Distinguishable Over a Wide Analyte Range. *Advanced Functional Materials* **23**, 4094-4104, doi:10.1002/adfm.201300245 (2013).

7 Wang, B. *et al.* A Highly Sensitive Diketopyrrolopyrrole-Based Ambipolar Transistor for Selective Detection and Discrimination of Xylene Isomers. *Advanced materials* **28**, 4012-4018, doi:10.1002/adma.201505641 (2016).

8 Wang, B., Sonar, P., Manzhos, S. & Haick, H. Diketopyrrolopyrrole copolymers based chemical sensors for the detection and discrimination of volatile organic compounds. *Sensors and Actuators B: Chemical* **251**, 49-56, doi:https://doi.org/10.1016/j.snb.2017.04.167 (2017).
